# Combination of 5‐aminolevulinic acid and ferrous ion reduces plasma glucose and hemoglobin A1c levels in Zucker diabetic fatty rats

**DOI:** 10.1002/2211-5463.12048

**Published:** 2016-04-29

**Authors:** Takeshi Hara, Aya Koda, Naoko Nozawa, Urara Ota, Hikaru Kondo, Hitoshi Nakagawa, Atsuko Kamiya, Kazutoshi Miyashita, Hiroshi Itoh, Motowo Nakajima, Tohru Tanaka

**Affiliations:** ^1^ SBI Pharmaceuticals Co., Ltd.Minato‐kuTokyoJapan; ^2^ Department of Internal MedicineSchool of MedicineKeio UniversityTokyoJapan

**Keywords:** 5‐aminolevulinic acid, heme oxygenase‐1, mitochondria, sodium ferrous citrate, type 2 diabetes mellitus, Zucker diabetic fatty rat

## Abstract

Mitochondrial dysfunction is associated with type 2 diabetes mellitus (T2DM). 5‐Aminolevulinic acid (ALA), a natural amino acid produced only in the mitochondria, is a precursor of heme. Cytochromes that contain heme play an important role in aerobic energy metabolism. Thus, ALA may help reduce T2DM‐associated hyperglycemia. In this study, we investigated the effect of ALA combined with sodium ferrous citrate (SFC) on hyperglycemia in Zucker diabetic fatty (ZDF) rats. We found that the gavage administration of ALA combined with SFC (ALA/SFC) for 6 weeks reduced plasma glucose and hemoglobin A1c (HbA1c) levels in rats without affecting plasma insulin levels. The glucose‐lowering effect depended on the amount of ALA/SFC administered per day. Furthermore, the glucose tolerance was also significantly improved by ALA/SFC administration. Although food intake was slightly reduced in the rats administered ALA/SFC, there was no effect on their body weight. Importantly, ALA/SFC administration induced heme oxygenase‐1 (HO‐1) expression in white adipose tissue and liver, and the induced expression levels of HO‐1 correlated with the glucose‐lowering effects of ALA/SFC. Taken together, these results suggest that ALA combined with ferrous ion is effective in reducing hyperglycemia of T2DM without affecting plasma insulin levels. HO‐1 induction may be involved in the mechanisms underlying the glucose‐lowering effect of ALA/SFC.

**Abbreviations**
ALA: 5‐Aminolevulinic acid
AUC: Area under the curve
BAT: Brown adipose tissue
HbA1c: Hemoglobin A1c
HO‐1: Heme oxygenase‐1
OGTT: Oral glucose tolerance test
OLETF: Otsuka Long‐Evans Tokushima Fatty
PCR: Polymerase chain reaction
PpIX: Protoporphyrin IX
ROS: Reactive oxygen species
SFC: Sodium ferrous citrate
T2DM: Type 2 diabetes mellitus
WAT: White adipose tissue
ZDF: Zucker diabetic fatty

Type 2 diabetes mellitus (T2DM) is a major metabolic disease characterized by both fasting and postprandial hyperglycemia 1. T2DM involves reduced insulin secretion due to failure of pancreatic β‐cell function and insulin resistance in the target organs, such as the adipose tissue, skeletal muscle, and liver 1. In 2010, approximately 310 million people were diagnosed with T2DM worldwide 2, and this number is expected to increase in the future 1. T2DM is known to cause various complications such as cardiovascular diseases and retinopathy 3, and the severity of these complications is closely linked to the risks of morbidity and mortality. Thus, the worldwide epidemic of T2DM represents a major health care problem in the 21st century.

Multiple factors, including genetic factors, physical inactivity, and changes in food intake, in combination with a sedentary lifestyle can lead to T2DM 4. Chronic overnutrition induces intracellular lipid accumulation in the peripheral tissues, which is associated with mitochondrial dysfunction 5. Mitochondrial dysfunction is linked to decreased ATP production and β‐oxidation and to accumulation of lipid intermediates and reactive oxidative species (ROS) 1, 6, 7. There is growing evidence that mitochondrial dysfunction is associated with T2DM 5, 8, 9, 10; for example, skeletal muscle biopsy samples from T2DM patients show that mitochondrial complex I activity was reduced by approximately 40% 9. Moreover, microarray analysis has indicated that the expression of the genes involved in mitochondrial oxidative metabolism is reduced in T2DM patients 8. In addition, the rates of mitochondrial oxidative phosphorylation in offspring of T2DM patients are reduced 10. Therefore, improvements in the mitochondrial activity, which can be induced by pharmacological drugs or supplements, may reduce hyperglycemia of T2DM.

5‐Aminolevulinic acid (ALA) is a natural amino acid and exists in all kinds of animals and plants 11. ALA is a common precursor of tetrapyrrole compounds including heme and chlorophylls and synthesized from glycine and succinyl CoA by mitochondrial ALA synthase in animal cells 11. The polymerization of eight molecules of ALA produces a precursor of heme, protoporphyrin IX (PpIX), which is used as a photosensitizer for photodynamic diagnosis and therapy to identify and kill tumor cells 11, 12, 13. Heme is generated by the insertion of ferrous ion into PpIX in the mitochondria 11. A recent study has suggested that ALA enhances aerobic energy metabolism, especially cytochrome *c* oxidase activity and protein expression in the mitochondria 14. In addition, abnormal heme biosynthesis can cause porphyria cutanea tarda and is often associated with T2DM 15. Furthermore, ALA has been demonstrated to induce heme oxygenase‐1 (HO‐1) expression in the kidney as well as in cultured cells 16, 17, 18. HO‐1 is a rate‐limiting enzyme in heme metabolism 11, and the upregulation of HO‐1 generates cytoprotective products such as bilirubin and carbon monoxide 19. Interestingly, increased intracellular heme levels lead to upregulation of HO‐1 expression 20, and HO‐1 has been shown to play a role in reducing hyperglycemia in several diabetes models 21, 22, 23.

There are two previous large‐scale intervention studies in which ALA combined with sodium ferrous citrate (ALA/SFC) was administered to prediabetes volunteers 24, 25. Rodriguez *et al*. 24 reported that the oral administration of ALA/SFC for 12 weeks improved the glucose levels of 2‐h postoral glucose tolerance test (OGTT) in prediabetes volunteers in Hawaii. These improved levels were particularly noticeable in prediabetes subjects with 2‐h post‐OGTT baseline levels of ≥ 140 mg·dL^−1^ (7.8 mmol·L^−1^), who displayed significantly better glucose‐lowering effects compared to those with baseline levels of < 140 mg·dL^−1^. Higashikawa *et al*. 25 also studied subjects with mild hyperglycemia selected from the general population in Hiroshima, Japan, and reported that the oral administration of ALA/SFC for 12 weeks reduced the fasting and 2‐h post‐OGTT plasma glucose levels without adverse events. These reports suggest that ALA/SFC improves glucose tolerance in prediabetes subjects. However, how ALA combined with ferrous ion reduces hyperglycemia of T2DM remains to be clarified.

As ALA combined with ferrous ion effectively induces the production of heme 26, we examined the effects of ALA/SFC on the plasma blood glucose and hemoglobin A1c (HbA1c) levels using Zucker diabetic fatty (ZDF) rats, which is a well‐known diabetes animal model. Furthermore, we examined the effects of ALA/SFC on the plasma insulin levels, pancreatic β‐cell mass, and HO‐1 expression in various tissues in order to explore possible mechanisms of the reduction in plasma glucose level by ALA/SFC in diabetic conditions.

## Materials and methods

### Chemicals

5‐Aminolevulinic acid hydrochloride (lot number: HCL‐KK08‐04‐1‐1; purity: 99.8%) was obtained from Cosmo Oil Co., Ltd (Tokyo, Japan). SFC was purchased from Komatsuya Corporation (Osaka, Japan). Pioglitazone was purchased from Pfizer Japan, Inc. (Tokyo, Japan).

### Animals

The animal study was conducted at Tsukuba Research Institute, BoZo Research Center Inc., Japan. Seven‐week‐old male ZDF rats (ZDF/Crl‐Lepr^fa^; fa/fa) were purchased from Charles River Laboratories Japan (Yokohama, Japan) and fed a CRF‐1 diet (Oriental Yeast, Tokyo, Japan). All rats were maintained on a 12‐h light‐dark cycle and given free access to food and water except during the period of caloric restriction for the pair‐feeding group. This study was approved by the Institutional Animal Care and Use Committee of Tsukuba Research Institute, BoZo Research Center Inc., and was performed according to the institutional guidelines on the management and welfare of laboratory animals.

### ALA/SFC administration

**Figure 1:**
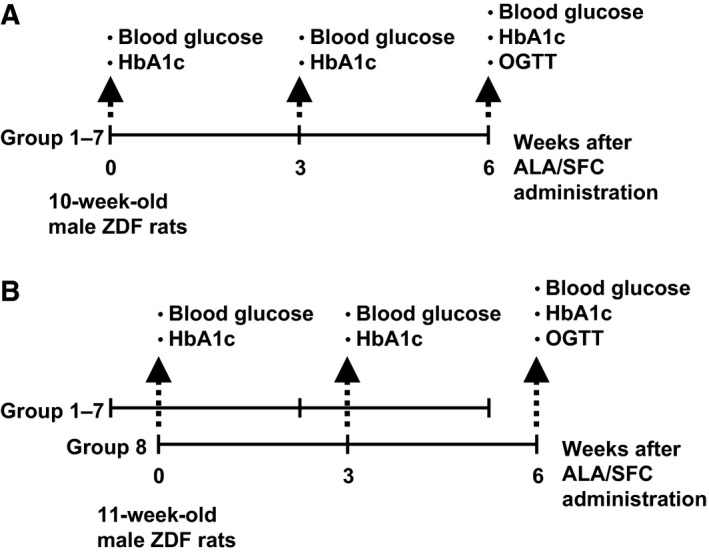
Experimental schedule. The experimental schedules for groups 1–7 (A) and group 8 (pair‐feeding group, B) are shown. The ZDF rats were orally administered the experimental treatment for 6 weeks. The plasma glucose and HbA1c levels were measured before the start of administration (0 weeks) and after 3 and 6 weeks. An OGTT was conducted 2–3 days after the 6‐week blood sampling. After the OGTT, on the same day, the rats underwent necropsy. Group 8 underwent vehicle administration 1 week after the start of administration of the first set. They were given the same amount of food once daily as the rats administered 300/47.1 mg·kg^−1^ ALA/SFC (group 7) in the previous week. The number of rats in all groups is eight.

All experiments involving ALA/SFC administration to the ZDF rats were performed at the same time. During the acclimation period, the ZDF rats were divided (Table 1) to match the average plasma glucose levels, HbA1c levels, body weight, and food intake. ALA, SFC, and pioglitazone were dissolved in distilled water for injection (Otsuka Pharmaceutical Factory, Inc., Tokushima, Japan). Ten‐week‐old (groups 1–7, set 1) or 11‐week‐old (group 8, set 2) ZDF rats were orally administered ALA/SFC or a vehicle by gavage for 6 weeks (Fig. 1). The dosing volume was 10 mL·kg^−1^, and the molar ratio of ALA to ferrous ion was 1:0.05. The rats were administered ALA/SFC once daily except for rats administered 150/23.6 mg·kg^−1^ ALA/SFC twice daily. The control pair‐feeding group (group 8) was administered the vehicle 1 week after the start of administration of the first set, and were administered the same amount of food (once daily) as the rats administered 300/47.1 mg·kg^−1^ ALA/SFC (group 7) in the previous week. Time course blood sampling, measurement of body weight, OGTT, and necropsy were also performed 1 week after the first set. ALA/SFC was administered between 09:00 and 12:00 in all rats. The second administration was performed > 6 h after the first dosing.

**Table 1:** Summary of the experimental groups

Group	Compound	Dose (mg·kg^−1^)	*n*
1	Vehicle	–	8
2	Pioglitazone	30	8
3	ALA/SFC	100/15.7	8
4	ALA/SFC	150/23.6	8
5	ALA/SFC (twice daily)	150/23.6	8
6	ALA/SFC	200/31.4	8
7	ALA/SFC	300/47.1	8
8 (pair‐feeding)	Vehicle	–	8

Rats were administered ALA/SFC once daily except for rats administered 150/23.6 mg·kg^−1^ ALA/SFC twice daily (group 5). Group 8 is the pair‐feeding group, which received the vehicle 1 week after the start of administration of the first set (groups 1–7). Group 8 was given the same amount of food once daily as group 7 in the previous week. The number of rats in all groups is eight. The molar ratio of ALA to ferrous ion was 1:0.05. All ZDF rats survived for the duration of the ALA/SFC administration.

### Measurement of body weight and food intake

Body weight was measured at least once a week, as well as on each starting day of administration, just before dosing. Food intake was measured once or twice a week. If the food intake was measured twice a week, the food weight was measured at 3–4 day intervals, and the daily food intake was calculated from the difference.

### Biochemical analyses

Blood sampling was performed in the morning, after removal of food for 1–2 h. Blood samples were transferred to microfuge tubes and centrifuged at 800 ***g*** at room temperature for 5 min. The hematocyte fractions were used for measurements of the HbA1c levels. The plasma was obtained and centrifuged again at 2000 ***g*** at room temperature for 10 min and used for measurements of the plasma glucose and insulin levels. The plasma glucose levels were determined by the glucose oxidase method using CicaLiquid GLU (Kanto Chemical, Tokyo, Japan). The HbA1c levels were estimated by an enzymatic method using Norudia N HbA1c (Sekisui Medical Co., Ltd., Tokyo, Japan). The plasma insulin concentration was determined using a rat insulin enzyme‐linked immunosorbent assay kit (Morinaga Institute of Biological Science, Inc., Kanagawa, Japan).

### Oral glucose tolerance test

An OGTT was conducted 2–3 days after the 6‐week blood sampling. After the last ALA/SFC administration, the rats were fasted overnight. On the day of the test, the body weight was measured and blood samples were taken from the tail vein using heparinized capillary tubes. Glucose (2 g glucose/10 mL·kg^−1^) was subsequently administered orally, and blood samples were collected at 15, 30, 60, 90, and 120 min after glucose administration.

### Measurement of pancreatic β‐cell mass

After the OGTT, on the same day, the rats underwent necropsy. The pancreatic β‐cell mass was measured as follows: the pancreas was fixed with paraformaldehyde solution and then embedded in paraffin. Five sections from the head to the tail of the pancreas were created and stained with anti‐insulin antibody (Dako, Kyoto, Japan). Using a microscope equipped with a 3CCD digital camera (Olympus Corporation, Tokyo, Japan) and image analysis software (FLVFS‐LS ver. 1.12; Flovel, Tokyo, Japan), the β‐cell area per total pancreatic area was measured (at Gotemba Laboratory, BoZo Research Center Inc., Shizuoka, Japan). The mean areas of the five sections were calculated for each animal, and the β‐cell mass per pancreas was calculated using the following formula:

β‐cell mass per pancreas (mg) = average β‐cell area per total pancreatic area × pancreatic weight

### Total RNA extraction and real‐time polymerase chain reaction

Total RNA was isolated from a tissue sample taken at necropsy for gene expression analysis using an RNA extraction kit (RNeasy Plus Universal Mini Kit, Qiagen, Tokyo, Japan) according to the manufacturer's instructions. Quantitative and qualitative analyses were performed using an Experion automated electrophoresis system (Bio‐Rad Laboratories, Hercules, CA, USA). cDNA was synthesized using the SuperScript VILO cDNA Synthesis Kit (Life Technologies, Carlsbad, CA, USA). The expression levels of HO‐1 mRNA were measured with TaqMan Gene Expression Assays (Life Technologies, Rn01536933_m1) using the 7500FAST real‐time polymerase chain reaction (PCR) system (Life Technologies). One of the cDNA samples was serially diluted and analyzed in parallel to obtain standard curves. The expression levels of all samples were normalized according to the expression levels of an endogenous gene control, β‐actin.

### Immunoblotting

Liver or epididymal fat was lysed in RIPA buffer (182‐02451; Wako Pure Chemicals, Tokyo, Japan) containing 1% Halt Protease Inhibitor Cocktail (100×) for 1 h on ice. After centrifugation (20 400 ***g***, 20 min, 4 °C) twice, the protein concentration in the supernatant was measured by Pierce^™^ BCA Protein Assay Kit (Thermo Fisher Scientific, Rockford, IL, USA) and then the supernatant was diluted with SDS sample buffer. Twenty microgram of liver protein or 16 μg of epididymal fat protein were loaded per lane, fractionated using 4–15% Mini‐PROTEAN® TGX^™^ Gel (456‐1085, Bio‐Rad Laboratories), and electrotransferred onto a Trans‐Blot® Turbo^™^ Mini PVDF Transfer Pack (170‐4156, Bio‐Rad Laboratories) by Trans**‐**Blot Turbo transfer system (Bio‐Rad Laboratories). Then the membranes were treated with 5% nonfat milk (Wako Pure Chemicals) in TBST (50 mm Tris‐HCl pH7.6, 150 mm NaCl and 0.1% Tween 20) for 1 h at room temperature, followed by incubation with antisera raised against HO‐1 (kindly provided from Dr. Shigeru Taketani, Department of Biotechnology, Kyoto Institute of Technology) or anti‐β‐actin antibody (ab8227; Abcam, Cambridge, UK) at 4 °C overnight. Furthermore, the membranes were washed three times for 10 min with TBST, incubated with HRP‐linked anti‐rabbit IgG antibody (NA934; GE Healthcare, Piscataway, NJ, USA) for 1 h at room temperature and washing three times with TBST for 10 min. Then specific protein was visualized with Immuno Star LD (292‐69903; Wako Pure Chemicals) using ChemiDoc MP system (Bio‐Rad Laboratories).

### Statistical analysis

All results are expressed as the mean ± SE. Statistical differences were analyzed using one‐way analysis of variance followed by Tukey's test (except for the data in Fig. 7C). For the Fig. 7C, statistical significance was determined using a two‐tailed unpaired Student's *t*‐test. *P*‐values < 0.05 were considered statistically significant.

## Results

### ALA/SFC reduces food intake, but does not affect body weight

**Figure 2:**
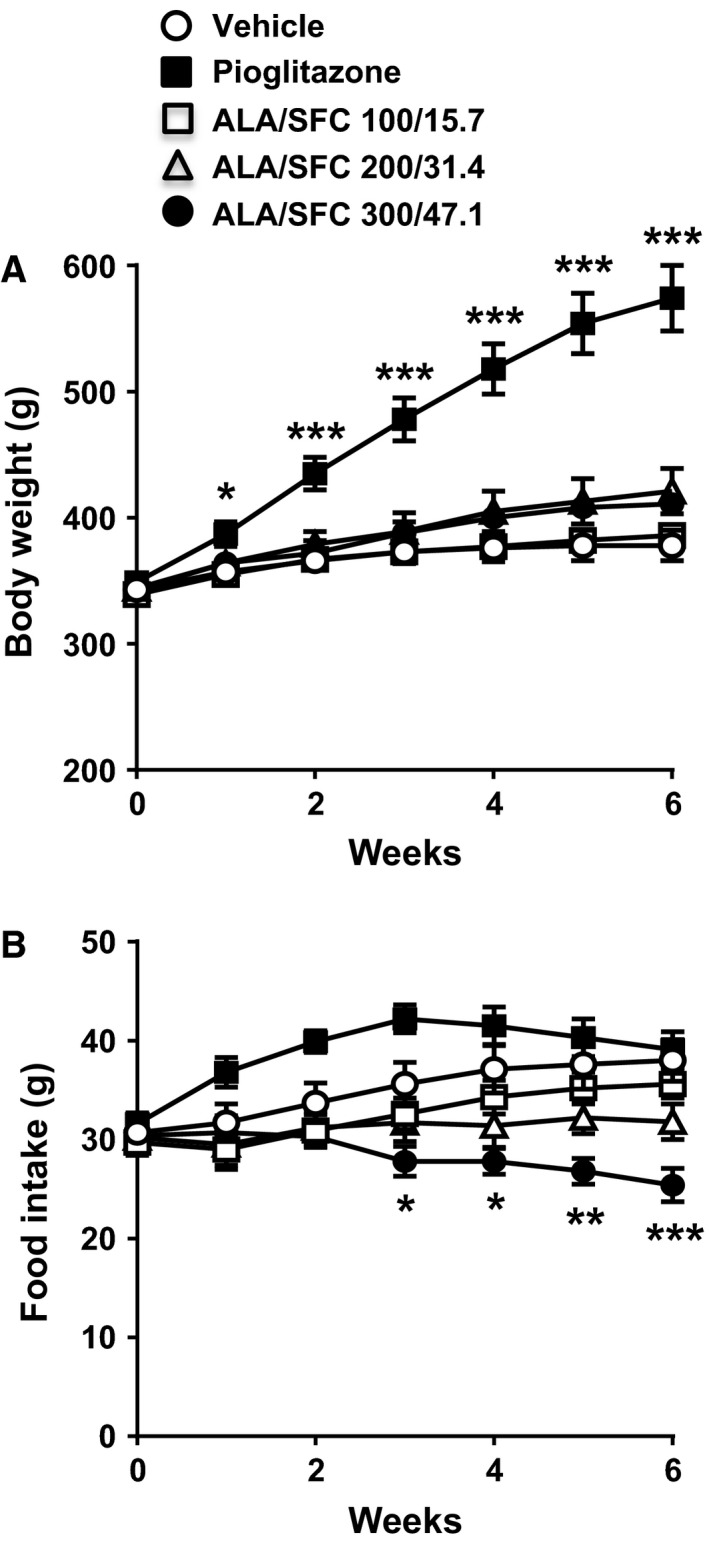
ALA/SFC reduces food intake, but does not affect body weight in ZDF rats. The ZDF rats were orally administered ALA/SFC or pioglitazone (30 mg·kg^−1^) for 6 weeks. (A) Body weight. (B) Food intake. The mean values per week are shown. *P < 0.05, **P < 0.01, ***P < 0.001 vs the vehicle‐administered group (Tukey's test, *n* = 8).

We examined the effects of ALA/SFC on body weight and food intake in ZDF rats. A previous report showed that the administration of ALA/SFC to Otsuka Long‐Evans Tokushima Fatty (OLETF) rats for 6 weeks reduced plasma glucose levels and improved impaired glucose tolerance 27. Thus, we used the maximum doses of 300/47.1 mg·kg^−1^ ALA/SFC. During administration of ALA/SFC for 6 weeks, the body weight of the ZDF rats slightly increased, but there was no significant difference between the vehicle‐administered and ALA/SFC‐administered rats (Fig. 2A). The rats administered pioglitazone, as a positive control, displayed a marked increase in body weight.

During administration of ALA/SFC, the food intake in rats administered 200/31.4 and 300/47.1 mg·kg^−1^ ALA/SFC decreased compared with that in rats administered the vehicle (Fig. 2B). At 6 weeks, the food intake in rats administered 300/47.1 mg·kg^−1^ ALA/SFC significantly decreased. However, the food intake in rats administered 200/31.4 mg·kg^−1^ ALA/SFC did not significantly differ from that in vehicle‐administered rats (*P* = 0.11). These data indicate that ALA/SFC reduces food intake, but does not affect body weight in ZDF rats.

### ALA/SFC reduces blood glucose and HbA1c levels

**Figure 3:**
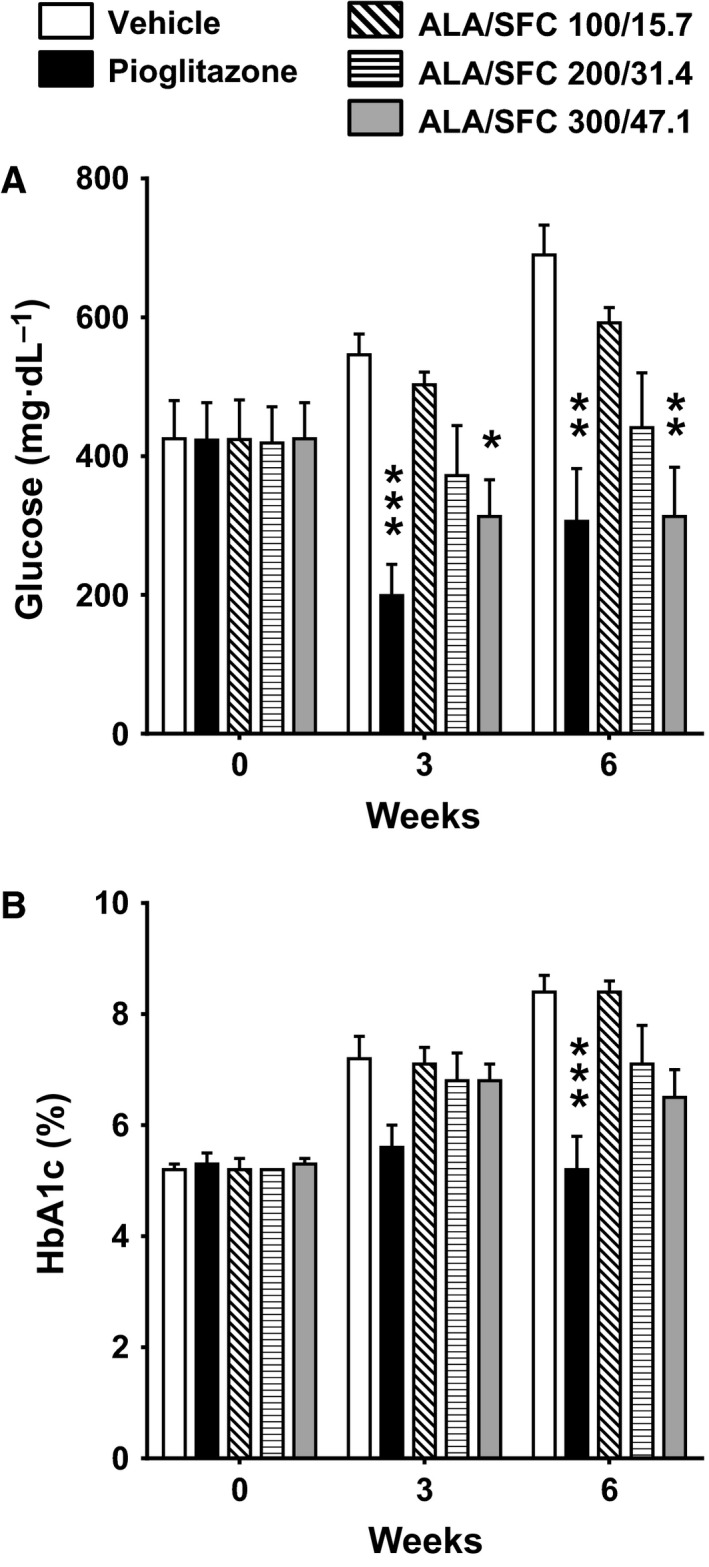
ALA/SFC reduces blood glucose and HbA1c levels in ZDF rats. The ZDF rats were orally administered ALA/SFC or pioglitazone for 6 weeks. (A) Plasma glucose levels. (B) HbA1c levels. *P < 0.05, **P < 0.01, ***P < 0.001 vs the vehicle‐administered group (Tukey's test, *n* = 8).

Next, we investigated the effect of ALA/SFC on the plasma glucose and HbA1c levels in ZDF rats. As shown in Fig. 3A, the administration of 300/47.1 mg·kg^−1^ ALA/SFC once daily for 6 weeks significantly reduced the plasma glucose levels (*P* = 0.0012 vs the vehicle). The administration of 200/31.4 mg·kg^−1^ ALA/SFC seemingly reduced the plasma glucose levels, but the difference was not significant (*P* = 0.056 vs the vehicle). At 3 weeks, the administration of 300/47.1 mg·kg^−1^ ALA/SFC reduced the plasma glucose levels compared to that of vehicle (*P* = 0.012). The effects of 30 mg·kg^−1^ pioglitazone and 300/47.1 mg·kg^−1^ ALA/SFC were almost the same at 6 weeks (*P* > 0.9). In addition, as shown in Fig. 3B, the HbA1c levels in the rats administered 300/47.1 mg·kg^−1^ ALA/SFC were reduced at 6 weeks compared with those in rats administered the vehicle; however, the difference was not significant (*P* = 0.065, vs the vehicle, Tukey's test). When Dunnett's test was used, the HbA1c levels in the rats administered 300/47.1 mg·kg^−1^ ALA/SFC were significantly reduced (*P* = 0.013).

### The glucose‐lowering effect of ALA/SFC is not a secondary effect of reduction in food intake

**Figure 4:**
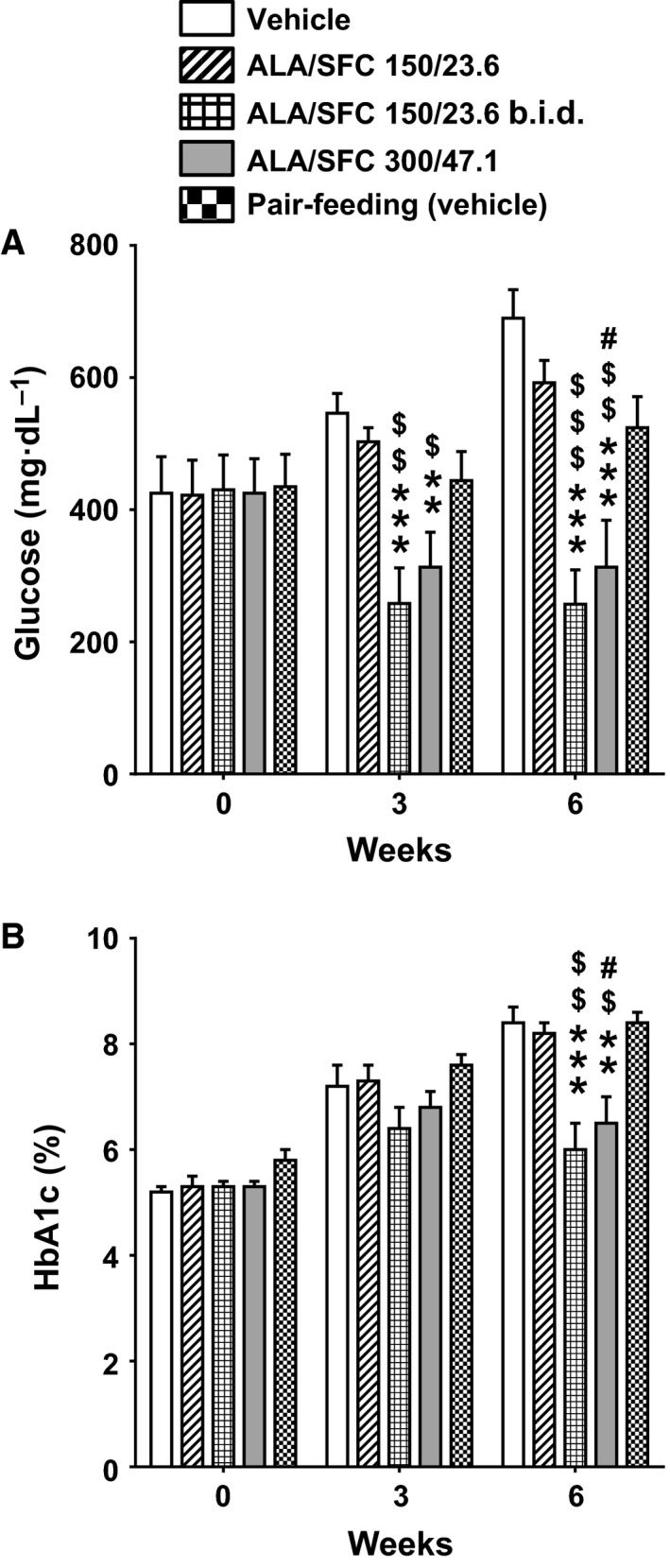
The glucose‐lowering effect of ALA/SFC, which is not a secondary effect due to reduced food intake, depends on the amount of ALA/SFC administered per day. ZDF rats were orally administered 150/23.6 or 300/47.1 mg·kg^−1^ ALA/SFC once a day or 150/23.6 mg·kg^−1^ ALA/SFC twice a day (b.i.d.) for 6 weeks. In addition, the pair‐feeding group (group 8) was given the same amount of food once daily as that given to rats administered 300/47.1 mg·kg^−1^ ALA/SFC in the previous week (group 7). (A) Plasma glucose levels. (B) HbA1c levels. **P < 0.01, ***P < 0.001 vs the vehicle‐administered group. ^#^ *P* < 0.05 vs the pair‐feeding group. ^$^ *P* < 0.05, ^$$^ *P* < 0.01, ^$$$^ *P* < 0.001 vs the 150/23.6 mg·kg^−1^ ALA/SFC group (Tukey's test, *n* = 8).

Because ALA/SFC administration appeared to the affect food intake in ZDF rats, as shown in Fig. 2B, pair‐feeding was conducted to examine the extent to which food intake reduction by ALA/SFC contributed to the plasma glucose‐lowering effect. Food intake in the pair‐feeding rats without ALA/SFC administration was matched to that in rats administered 300/47.1 mg·kg^−1^ ALA/SFC. The plasma glucose levels in the pair‐feeding rats were slightly, but not significantly, lower than those in the ad‐lib feeding vehicle‐administered rats (Fig. 4A). In addition, the plasma glucose levels in rats administered 300/47.1 mg·kg^−1^ ALA/SFC were significantly reduced compared with those in the pair‐feeding rats. The HbA1c levels in the pair‐feeding rats were not significantly different from those in the vehicle‐administered rats (Fig. 4B), while the HbA1c levels in rats administered 300/47.1 mg·kg^−1^ ALA/SFC were decreased significantly compared with that in the pair‐feeding rats (*P* = 0.010). These results suggest that the glucose‐lowering effect of ALA/SFC in ZDF rats is not a secondary effect of the reduction in food intake.

### The glucose‐lowering effect depends on the amount of ALA/SFC administered per day

Subsequently, the effects of once‐ or twice‐daily administration of ALA/SFC on the plasma glucose levels and HbA1c were investigated. The plasma glucose and HbA1c levels were slightly reduced in ZDF rats administered 150/23.6 mg·kg^−1^ ALA/SFC once daily, but were not significantly different from those in rats administered the vehicle (*P* > 0.7) (Fig. 4). However, the plasma glucose and HbA1c levels in the rats administered 150/23.6 mg·kg^−1^ ALA/SFC twice daily were markedly reduced (*P* < 0.001 vs the vehicle) at 6 weeks, and were almost the same as those observed with 300/47.1 mg·kg^−1^ ALA/SFC administration once daily (plasma glucose and HbA1c, *P* > 0.9) (Fig. 4). In addition, the plasma glucose and HbA1c levels in the rats administered 150/23.6 mg·kg^−1^ ALA/SFC twice daily were significantly decreased compared with those in the rats administered 150/23.6 mg·kg^−1^ ALA/SFC once daily (*P* < 0.001 and *P* < 0.01, respectively). These data suggest that the potency of the glucose‐lowering effect of ALA/SFC depends on the amount of ALA/SFC administered per day, and it might need more than 6 weeks to obtain a significant reduction in HbA1c level in ZDF rats administered 300/47.1 mg·kg^−1^ ALA/SFC total per day.

### ALA/SFC decreases fasting plasma glucose levels and improves glucose tolerance

**Figure 5:**
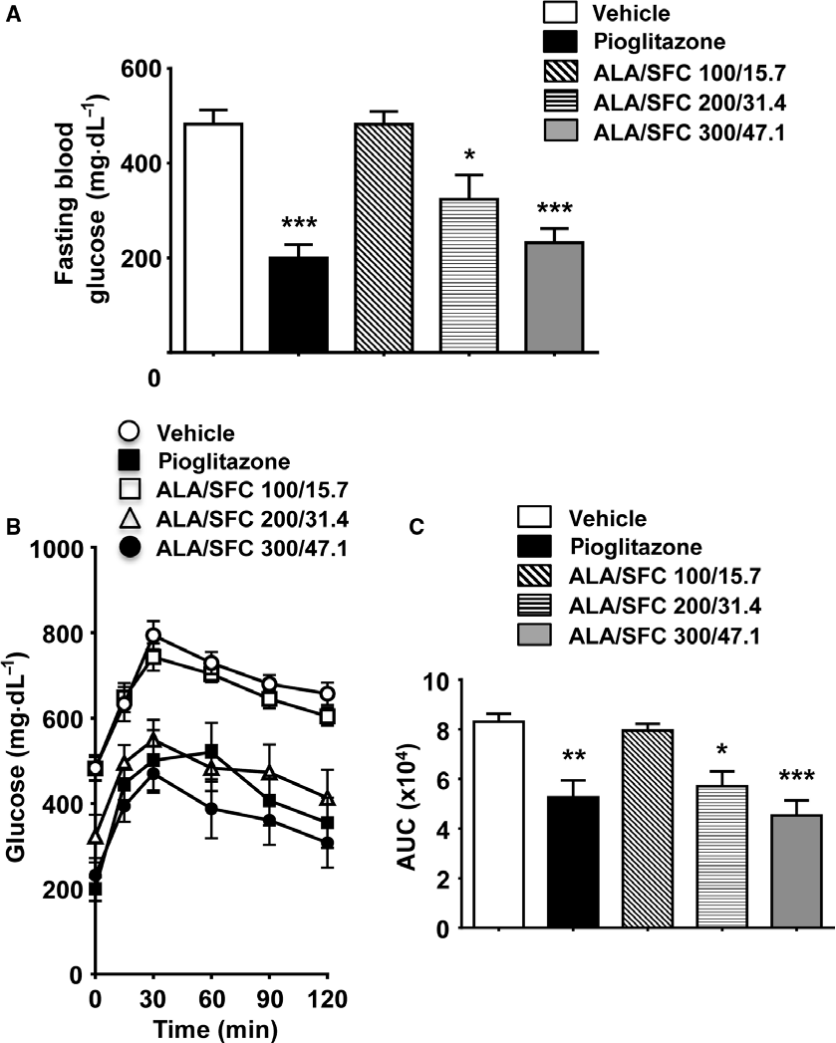
ALA/SFC decreases the fasting plasma glucose levels and improves glucose tolerance. An OGTT was conducted 2–3 days after the 6‐week blood sampling. After the last ALA/SFC administration, ZDF rats were fasted overnight, after which the OGTT was performed. (A) Fasting plasma glucose levels. (B) Plasma glucose levels in the OGTT. C. Area under the curve (AUC) of glucose levels in the OGTT. *P < 0.05, **P < 0.01, ***P < 0.001 vs the vehicle‐administered group (Tukey's test, *n* = 8).

Oral glucose tolerance tests were performed to determine whether administration of ALA/SFC for 6 weeks improves glucose tolerance in ZDF rats. The fasting plasma glucose levels were found to be significantly reduced by administration of both 200/31.4 and 300/47.1 mg·kg^−1^ ALA/SFC (Fig. 5A). Pioglitazone also reduced the levels of fasting plasma glucose. In the OGTT, the glucose tolerance was significantly improved by administration of 200/31.4 and 300/47.1 mg·kg^−1^ ALA/SFC (Fig. 5B). Consistently, the area under the curve (AUC) of the glucose levels significantly was decreased by administration of 200/31.4 and 300/47.1 mg·kg^−1^ ALA/SFC compared to rats administered the vehicle (Fig. 5C). The AUC of the glucose levels was also significantly decreased by pioglitazone. These data indicate that ALA/SFC decreases the fasting plasma glucose and improves glucose tolerance.

### Administration of ALA/SFC does not affect plasma insulin levels

**Figure 6:**
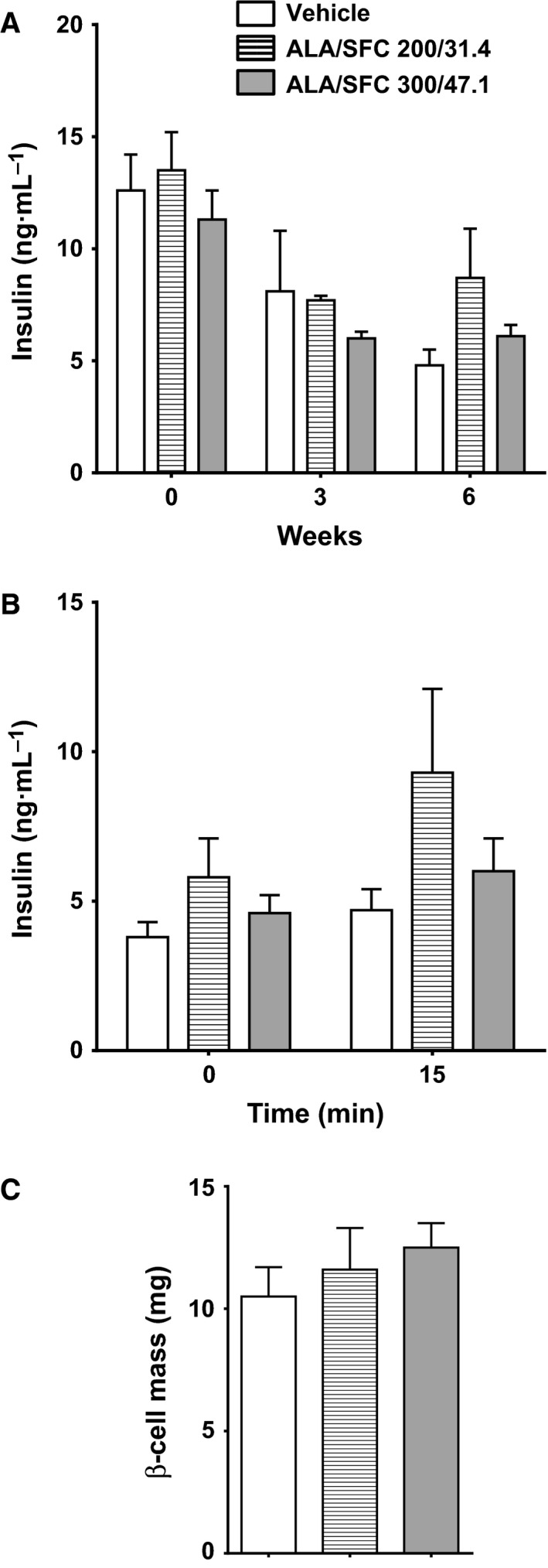
ALA/SFC does not affect the plasma insulin levels in ZDF rats. (A) Plasma insulin levels during 6‐week administration of ALA/SFC. (B) Plasma insulin levels in the OGTT after 6‐week administration of ALA/SFC. The OGTT was conducted 2–3 days after the 6‐week blood sampling. After the last ALA/SFC administration, the rats were fasted overnight, after which the OGTT was performed. The insulin levels were measured before glucose administration and at 15 min. (C) Pancreatic β‐cell mass after 6‐week administration of ALA/SFC.

We moreover examined the plasma insulin levels before administering ALA/SFC and after 3 and 6 weeks. The plasma insulin levels in rats administered 200/31.4 and 300/47.1 mg·kg^−1^ ALA/SFC were not significantly different from those in rats administered the vehicle (Fig. 6A). The plasma insulin levels were also measured during the OGTT; the fasting plasma insulin levels of rats administered 200/31.4 and 300/47.1 mg·kg^−1^ ALA/SFC were not significantly different from those of the rats administered the vehicle. At 15 min after glucose administration, there were no significant differences in the insulin levels among all groups (Fig. 6B). Furthermore, the average β‐cell mass of rats administered 200/31.4 or 300/47.1 mg·kg^−1^ ALA/SFC was not significantly different from that of the rats administered the vehicle (Fig. 6C). Thus, these data suggest that ALA/SFC does not affect the plasma insulin levels and β‐cell mass in ZDF rats.

### ALA/SFC increases HO‐1 expression in white adipose tissue and the liver

**Figure 7:**
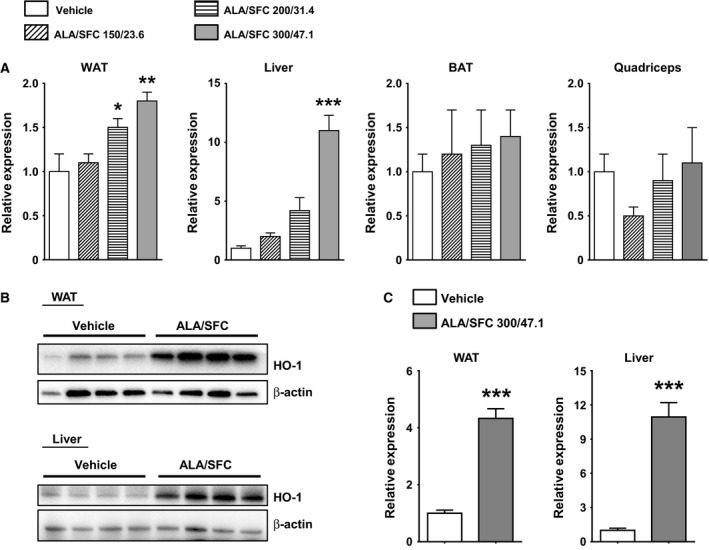
Expression levels of HO‐1 in various tissues of ZDF rats administered ALA/SFC. (A) Expression levels of HO‐1 mRNA in tissues after 6‐week administration of ALA/SFC. The expression levels are shown as values relative to those of the vehicle‐administered group (Set to 1.0). *P < 0.05, **P < 0.01, ***P < 0.001 vs the vehicle‐administered group (Tukey's test, *n* = 4). (B) The expression levels of HO‐1 protein in the liver and WAT of the rats administered vehicle or 300/47.1 mg·kg^−1^ ALA/SFC were analyzed by immunoblotting. (C) The relative expression levels of HO‐1 protein to β‐actin. The expression levels of 300/47.1 mg·kg^−1^ ALA/SFC‐administered group are shown as relative values compared to those of the vehicle‐administered group (Set to 1.0). ***P < 0.001 vs the vehicle‐administered group (Student's *t*‐test, *n* = 4).

Increased intracellular heme levels have been shown to lead to upregulation of HO‐1 expression 20, and HO‐1 is known to play an important role in reducing hyperglycemia in several diabetes animal models 21, 22, 23. Thus, we examined the expression levels of HO‐1 in various tissues, including the epididymal white adipose tissue (WAT), brown adipose tissue (BAT), quadriceps muscle, and liver. In the liver, the HO‐1 mRNA levels were significantly higher in rats administered 300/47.1 mg·kg^−1^ ALA/SFC compared with those administered the vehicle (Fig. 7A). In the epididymal WAT, the HO‐1 mRNA levels were significantly higher in rats administered 200/31.4 and 300/47.1 mg·kg^−1^ ALA/SFC compared with those administered the vehicle (Fig. 7A). However, the HO‐1 mRNA levels in rats administered 150/23.6 mg·kg^−1^ ALA/SFC were not significantly different from those in rats administered the vehicle. The induced expression of HO‐1 correlated with the glucose‐lowering effect of ALA/SFC. There were no significant differences in the HO‐1 expression levels in the BAT and quadriceps muscle between the vehicle‐administered and ALA/SFC‐administered rats. In addition, the expression levels of HO‐1 proteins in the liver and WAT of the rats administered 300/47.1 mg·kg^−1^ were higher than those in the vehicle (Fig. 7B and 7C). Accordingly, these data suggest that ALA/SFC upregulates HO‐1 expression in the epididymal WAT and liver of ZDF rats.

## Discussion

In this study, we examined the effects of ALA/SFC on plasma blood glucose and HbA1c levels using ZDF rats. We found that ALA/SFC administration reduced the plasma glucose and fasting blood glucose levels. These effects were in an apparent dose‐dependent manner. Furthermore, ALA/SFC administration reduced the HbA1c levels. ALA/SFC also reduced the food intake, but did not affect the body weight of the rats. In addition, ALA/SFC improved glucose tolerance in an apparent dose‐dependent manner, which is consistent with the results of the previous study that found that administration of 300/47.1 mg·kg^−1^ ALA/SFC for 6 weeks in OLETF rats improved glucose tolerance 27. Finally, ALA/SFC increased HO‐1 expression in the WAT and liver. Taken together, these findings indicate that ALA, which is an endogenous δ‐amino acid, may be effective in reducing hyperglycemia of T2DM.

We considered a possible mechanism underlying the glucose‐lowering effect of ALA/SFC. HO‐1 has antioxidant activity through bilirubin production, which leads to decreased ROS and consequently protects against oxidative stress‐induced cell injury in various tissues including the liver 19, 28, 29. A decrease in the accumulated intracellular ROS is associated with enhanced mitochondrial function and insulin signaling 7, 30, 31, 32. In fact, upregulation of HO‐1 has been demonstrated to improve glucose metabolism and enhance insulin signaling in several diabetes animal models, which in turn is linked to antioxidant mechanisms 21, 22, 23. Furthermore, the HO‐1 mRNA expression was significantly reduced in T2DM patients, which is associated with dysfunction of the antioxidant defense mechanisms 33. Our findings show that ALA/SFC upregulates HO‐1 expression in the WAT and liver, which might be associated with decreased ROS, and induction of HO‐1 could be partially involved in the mechanism underlying the glucose‐lowering effect of ALA/SFC. ALA/SFC might induce HO‐1 expression by increasing the intracellular content of heme 34. Increased expression of HO‐1 protein is correlated with the HO activity 21, 35, 36. ALA combined with ferrous ion induces carbon monoxide, produced from heme through activation of HO‐1 in mouse kidney 37. This report suggests that ALA combined with ferrous ion induces HO‐1 activity. Thus, in the next step, the measurement of HO activity in the ZDF rats administered ALA/SFC would in principle be investigated. In addition, the role of increases in HO activity could be investigated by use of HO‐1 inhibitors such as zinc protoporphyrin. Furthermore, additional studies using HO‐1 knockout mice would be useful to clarify whether upregulation of HO‐1 is involved in the glucose‐lowering effect of ALA/SFC. The exact molecular mechanisms underlying the upregulation of HO‐1 by ALA/SFC remain to be elucidated.

Other mechanisms may be responsible for the glucose‐lowering effect of ALA/SFC. Rats fed a normal diet and administered a low‐dose ALA for 14 days display increased oxygen consumption and decreased WAT weight 38, suggesting that ALA may promote energy metabolism. Furthermore, ALA increases cytochrome *c* oxidase activity and enhances aerobic energy metabolism 14. In addition, ALA induces the expression of cytochrome *c* oxidase, which is one of the heme‐containing proteins 14. Thus, ALA/SFC may enhance mitochondrial bioactivity, which consequently increases production of ATP and NAD+, decreases ROS production, and upregulates oxidative phosphorylation and the tricarboxylic acid cycle. These metabolic changes can enhance the intracellular glucose consumption, which would result in increased glucose uptake in various tissues such as the adipose tissues, muscles, and liver. However, further studies are required to elucidate the mechanisms underlying the glucose‐lowering effect of ALA/SFC.

The glucose‐lowering effect of ALA/SFC might be due to the reduction of food intake. However, as shown in Fig. 4, the pair‐feeding rats did not show such a remarkable reduction in plasma glucose and HbA1c levels, suggesting that the glucose‐lowering effect of ALA/SFC is not a secondary effect of food intake. It has been reported that eating speed is important for glucose metabolism 39. Therefore, there is a possibility that ALA/SFC might reduce eating speed and thus lower the plasma glucose levels. In this study, we cannot rule out this possibility. The measurement of the precise eating speed of ZDF rats in each group would be required to answer this question.

In this study, there were two reasons why we used the doses of 300/47.1 mg·kg^−1^ ALA/SFC. First, the administration of 300/47.1 mg·kg^−1^ ALA/SFC to OLETF rats for 6 weeks reduced plasma glucose levels and improved impaired glucose tolerance 27. Second, in preliminary experiments, low dose of ALA such as 3 or 10 mg·kg^−1^ did not reduced plasma glucose levels in ZDF rats (unpublished data). However, the typical intake of ALA from food is approximately 1–2 mg·day^−1^ 40. Thus, ALA 300 mg·kg^−1^ would be pharmacologic dose, but not physiologic dose.

In this study, the plasma insulin levels of ALA/SFC‐administered ZDF rats were not different from those of vehicle‐administered rats. This finding is consistent with the results of the previous study in which prediabetes patients were administered ALA/SFC for 12 weeks, and the fasting insulin levels were not affected 25. Decreased pancreatic β‐cell mass is associated with development of T2DM 41, 42. Furthermore, pancreatic β‐cell mass would be associated with insulin secretion 41, 42, 43. In this study, there was no significant difference in the pancreatic β‐cell mass between ALA/SFC‐administered and vehicle‐administered rats. Thus, the glucose‐lowering effect of ALA/SFC might not be explained by regulation of plasma insulin level.

As shown in Fig. 2, ALA/SFC decreased food intake in ZDF rats; however, the body weight was not affected. We have considered several possibilities to explain these phenomena. First, gavage administration of ALA/SFC by using a sonde might have caused irritation of stomach, because the pH of ALA/SFC solution in water is low. The irritation of stomach could decrease food intake and also suppress motions, resulting in reduction in energy consumption. Second, ALA/SFC might enhance caloric efficiency, because ALA enhances aerobic energy metabolism 14. In this case, decreased food intake shall not affect body weight. Third, ALA/SFC might enhance intestinal absorption of nutrients, which results in decrease in food intake but no change in body weight. Although in this study we did not focus on the mechanisms by which ALA/SFC decreased food intake without a significant change in body weight, it would be interesting to examine these possibilities in the future studies. In addition, it is reported that GLP‐1 plays important roles in not only insulin secretion but also appetite 44. Therefore GLP‐1 might be involved in decreased food intake by ALA/SFC. Although we did not measured the plasma levels of GLP‐1, we found that the plasma levels of insulin in ZDF rats administrated ALA/SFC were not different from those in ZDF rats administered vehicle, implying that ALA/SFC does not upregulate GLP‐1 secretion. Thus, GLP‐1 may not account for decreased food intake in ZDF rats administered ALA‐SFC in this study.

Some studies have reported that ALA induces DNA damage in cultured tumor cells, which may induce cytotoxicity 45, 46. A secondary effect of this cytotoxicity in normal tissues may be responsible for the plasma glucose‐lowering effect of 300/47.1 mg·kg^−1^ ALA/SFC in ZDF rats. However, it is well known that PpIX preferentially accumulates in tumor cells but not in normal cells 11, 12, 13. In fact, because ALA administration leads to accumulation of PpIX in tumor cells 11, 12, 13, ALA is used for not only photodynamic diagnosis to identify tumor cells, but also used as a photodynamic therapy agent to induce cell death in tumors 11. This cell death in tumors is probably due to ROS generation by PpIX after exposure to specific wavelength light 47. Furthermore, PpIX can only induce DNA damage without light excitation in cultured tumor cells, through unknown mechanisms 48, 49, 50, 51. As heme is generated by insertion of ferrous ion into PpIX 11, the production of ferrous ion from ferric ion is important to reduce PpIX in normal cells; the presence of iron chelators alone in mouse skin promotes the accumulation of PpIX 52. In contrast, ferrous ion promotes a decrease in intracellular PpIX and increases the production of heme 26, 53. In a previous study, the administration of 300/47.1 mg·kg^−1^ ALA/SFC for 6 weeks in OLETF rats did not show any changes in the mucosal sucrose activity or protein content in the small intestine 27. In addition, there was no difference in the body weight between ZDF rats administered 300/47.1 mg·kg^−1^ ALA/SFC and rats administered the vehicle in this study. Furthermore, to exclude a possibility of the cytotoxicity of ALA/SFC, we measured the plasma levels of alanine aminotransferase (ALT) and aspartate aminotransferase (AST). The ALT and AST in ZDF rats administered 300/47.1 mg·kg^−1^ ALA/SFC were 405 ± 68 (IU·L^−1^) and 485 ± 42 (IU·L^−1^) (mean ± SE, *n* = 8), respectively, while those in ZDF rats administered the vehicle were 400 ± 153 and 371 ± 137, respectively. There was no significant difference between these two groups. Therefore, a dose of 300/47.1 mg·kg^−1^ ALA/SFC in rats is not cytotoxic to normal tissues. Collectively, we can hence conclude that the glucose‐lowering effect of ALA/SFC in ZDF rats is not a secondary effect of cytotoxicity.

On the basis of our findings, we conclude that administration of ALA/SFC for 6 weeks reduced the plasma glucose and HbA1c levels in ZDF rats. When the ZDF rat dose is converted to the human‐equivalent dose based on body surface area 54, the human‐equivalent dose appears high. However, administration of even low doses of ALA/SFC to prediabetic populations in USA and Japan for 12 weeks improved glucose tolerance effectively in the previous studies 24, 25, implying that much lower doses of ALA/SFC may be effective in humans compared to those in ZDF rats. Of note, there are species‐specific differences in iron metabolism. For example, the iron uptake, storage, and flux in humans are different from those in mice 55. Thus, the difference of the effective dose between rats and humans might be explained by the different effective concentrations of ALA combined with ferrous ion in the target issues in each species.

The circadian rhythm controls numerous physiological processes. These processes include the metabolic pathways such as those related to glucose and lipid metabolism 56, 57, 58, 59. Dysregulation of the circadian rhythm is a risk factor of metabolic syndromes such as obesity and T2DM, as well as of cardiovascular diseases 58, 60. There is increasing evidence that heme plays an important role in the circadian rhythm 59, 61, 62. As ALA is the sole precursor of heme, ALA has been speculated to affect the circadian rhythm through activation of the REV‐ERBα nuclear receptor 63. Thus, the difference of the effective dose in humans and rats might also be explained by differences in the heme biosynthesis and degradation, and in iron metabolism, which may all affect the circadian rhythm. Accordingly, it is important to determine the appropriate dose of ALA/SFC for diabetic patients in clinical trials.

## Conclusions

Our results demonstrate that ALA/SFC can effectively reduce hyperglycemia of T2DM. However, further studies are required to elucidate the mechanisms of the glucose‐lowering effect of ALA/SFC. Our findings indicate that ALA/SFC may be an effective antidiabetic drug, providing a glucose‐lowering effect. Because ALA/SFC reduces the fasting and postprandial glucose levels in prediabetes patients, oral administration of ALA/SFC may represent a new therapeutic approach for not only new‐onset T2DM, but also advanced T2DM in humans.

## Author contributions

All authors contributed to the study design, concept and interpretation of data. T.H., A.K., N.N., and H.K. contributed to the acquisition and analysis of the data. T.H., A.K., M.N., and T.T. prepared the manuscript. All authors reviewed and approved the final manuscript.

## References

[1] Patti ME and Corvera S (2010) The role of mitochondria in the pathogenesis of type 2 diabetes. Endocr Rev 31, 364–395. 20156986 10.1210/er.2009-0027 PMC3365846

[2] Hagberg CE , Mehlem A , Falkevall A , Muhl L , Fam BC , Ortsäter H , Scotney P , Nyqvist D , Samén E , Lu L *et al* (2012) Targeting VEGF‐B as a novel administration for insulin resistance and type 2 diabetes. Nature 490, 426–430. 23023133 10.1038/nature11464

[3] Forbes JM and Cooper ME (2013) Mechanisms of diabetic complications. Physiol Rev 93, 137–188. 23303908 10.1152/physrev.00045.2011

[4] Hu FB (2011) Globalization of diabetes: the role of diet, lifestyle, and genes. Diabetes Care 34, 1249–1257. 21617109 10.2337/dc11-0442 PMC3114340

[5] Pagel‐Langenickel I , Bao J , Pang L and Sack MN (2010) The role of mitochondria in the pathophysiology of skeletal muscle insulin resistance. Endocr Rev 31, 25–51. 19861693 10.1210/er.2009-0003 PMC2852205

[6] Bonnard C , Durand A , Peyrol S , Chanseaume E , Chauvin MA , Morio B , Vidal H and Rieusset J (2008) Mitochondrial dysfunction results from oxidative stress in the skeletal muscle of diet‐induced insulin‐resistant mice. J Clin Invest 118, 789–800. 18188455 10.1172/JCI32601 PMC2176186

[7] Kim JA , Wei Y and Sowers JR (2008) Role of mitochondrial dysfunction in insulin resistance. Circ Res 102, 401–414. 18309108 10.1161/CIRCRESAHA.107.165472 PMC2963150

[8] Patti ME , Butte AJ , Crunkhorn S , Cusi K , Berria R , Kashyap S , Miyazaki Y , Kohane I , Costello M , Saccone R *et al* (2003) Coordinated reduction of genes of oxidative metabolism in humans with insulin resistance and diabetes: potential role of PGC1 and NRF1. Proc Natl Acad Sci USA 100, 8466–8471. 12832613 10.1073/pnas.1032913100 PMC166252

[9] Ritov VB , Menshikova EV , He J , Ferrell RE , Goodpaster BH and Kelley DE (2005) Deficiency of subsarcolemmal mitochondria in obesity and type 2 diabetes. Diabetes 54, 8–14. 15616005 10.2337/diabetes.54.1.8

[10] Befroy DE , Petersen KF , Dufour S , Mason GF , de Graaf RA , Rothman DL and Shulman GI (2007) Impaired mitochondrial substrate oxidation in muscle of insulin‐resistant offspring of type 2 diabetic patients. Diabetes 56, 1376–1381. 17287462 10.2337/db06-0783 PMC2995532

[11] Ishizuka M , Abe F , Sano Y , Takahashi K , Inoue K , Nakajima M , Kohda T , Komatsu N , Ogura S and Tanaka T (2011) Novel development of 5‐aminolevurinic acid (ALA) in cancer diagnoses and therapy. Int Immunopharmacol 11, 358–365. 21144919 10.1016/j.intimp.2010.11.029

[12] Regula J , MacRobert AJ , Gorchein A , Buonaccorsi GA , Thorpe SM , Spencer GM , Hatfield AR and Bown SG (1995) Photosensitisation and photodynamic therapy of oesophageal, duodenal, and colorectal tumors using 5 aminolaevulinic acid induced protoporphyrin IX – a pilot study. Gut 36, 67–75. 7890239 10.1136/gut.36.1.67 PMC1382355

[13] Zenzen V and Zankl H (2003) Protoporphyrin IX‐accumulation in human tumor cells following topical ALA‐and h‐ALA‐application in vivo. Cancer Lett 202, 35–42. 14643024 10.1016/j.canlet.2003.07.001

[14] Ogura S , Maruyama K , Hagiya Y , Sugiyama Y , Tsuchiya K , Takahashi K , Abe F , Tabata K , Okura I , Nakajima M *et al* (2011) The effect of 5‐aminolevulinic acid on cytochrome *c* oxidase activity in mouse liver. BMC Res Notes 4, 66. 21414200 10.1186/1756-0500-4-66 PMC3068109

[15] Rossmann‐Ringdahl I and Olsson R (2005) Porphyria cutanea tarda in a Swedish population: risk factors and complications. Acta Derm Venereol 85, 337–341. 16191856 10.1080/00015550510033688

[16] Frank J , Lornejad‐Schäfer MR , Schöffl H , Flaccus A , Lambert C and Biesalski HK (2007) Inhibition of heme oxygenase‐1 increases responsiveness of melanoma cells to ALA‐based photodynamic therapy. Int J Oncol 31, 1539–1545. 17982681

[17] Hagiya Y , Adachi T , Ogura S , An R , Tamura A , Nakagawa H , Okura I , Mochizuki T and Ishikawa T (2008) Nrf2‐dependent induction of human ABC transporter ABCG2 and heme oxygenase‐1 in HepG2 cells by photoactivation of porphyrins: biochemical implications for cancer cell response to photodynamic therapy. J Exp Ther Oncol 7, 153–167. 18771089

[18] Quadri S , Jackson DW , Prathipati P , Dean C and Jackson KE (2012) Heme induction with delta‐aminolevulinic acid stimulates an increase in water and electrolyte excretion. Int J Hypertens 2012, Article ID 690973. 10.1155/2012/690973 PMC3270426 22315666

[19] Abraham NG and Kappas A (2005) Heme oxygenase and the cardiovascular‐renal system. Free Radic Biol Med 39, 1–25. 15925276 10.1016/j.freeradbiomed.2005.03.010

[20] Alam J , Shibahara S and Smith A (1989) Transcriptional activation of the heme oxygenase gene by heme and cadmium in mouse hepatoma cells. J Biol Chem 264, 6371–6375. 2703493

[21] Li M , Kim DH , Tsenovoy PL , Peterson SJ , Rezzani R , Rodella LF , Aronow WS , Ikehara S and Abraham NG (2008) Administration of obese diabetic mice with a heme oxygenase inducer reduces visceral and subcutaneous adiposity, increases adiponectin levels, and improves insulin sensitivity and glucose tolerance. Diabetes 57, 1526–1535. 18375438 10.2337/db07-1764

[22] Nicolai A , Li M , Kim DH , Peterson SJ , Vanella L , Positano V , Gastaldelli A , Rezzani R , Rodella LF , Drummond G *et al* (2009) Heme oxygenase‐1 induction remodels adipose tissue and improves insulin sensitivity in obesity‐induced diabetic rats. Hypertension 53, 508–515. 19171794 10.1161/HYPERTENSIONAHA.108.124701 PMC2745551

[23] Ndisang JF , Lane N , Syed N and Jadhav A (2010) Up‐regulating the heme oxygenase system with hemin improves insulin sensitivity and glucose metabolism in adult spontaneously hypertensive rats. Endocrinology 151, 549–560. 20016031 10.1210/en.2009-0471

[24] Rodriguez BL , Curb JD , Davis J , Shintani T , Perez MH , Apau‐Ludlum N , Johnson C and Harrigan RC (2012) Use of the dietary supplement 5‐aminiolevulinic acid (5‐ALA) and its relationship with glucose levels and hemoglobin A1C among individuals with prediabetes. Clin Transl Sci 5, 314–320. 22883608 10.1111/j.1752-8062.2012.00421.x PMC5439781

[25] Higashikawa F , Noda M , Awaya T , Tanaka T and Sugiyama M (2013) 5‐aminolevulinic acid, a precursor of heme, reduces both fasting and postprandial glucose levels in mildly hyperglycemic subjects. Nutrition 29, 1030–1036. 23759263 10.1016/j.nut.2013.02.008

[26] Mingone CJ , Gupte SA , Chow JL , Ahmad M , Abraham NG and Wolin MS (2006) Protoporphyrin IX generation from delta‐aminolevulinic acid elicits pulmonary artery relaxation and soluble guanylate cyclase activation. Am J Physiol Lung Cell Mol Physiol 291, L337–L344. 16899710 10.1152/ajplung.00482.2005

[27] Sato T , Yasuzawa T , Uesaka A , Izumi Y , Kamiya A , Tsuchiya K , Kobayashi Y , Kuwahata M and Kido Y (2014) Type 2 diabetic conditions in Otsuka Long‐Evans Tokushima Fatty rats are ameliorated by 5‐aminolevulinic acid. Nutr Res 34, 544–551. 25026922 10.1016/j.nutres.2014.04.013

[28] Asija A , Peterson SJ , Stec DE and Abraham NG (2011) Targeting endothelial cells with heme oxygenase‐1 gene using VE‐cadherin promoter attenuates hyperglycemia‐mediated cell injury and apoptosis. Biochim Biophys Acta 1813, 668–682. 17883332 10.1089/ars.2007.1804

[29] Yu J , Chu ES , Wang R , Wang S , Wu CW , Wong VW , Chan HL , Farrell GC and Sung JJ (2010) Heme oxygenase‐1 protects against steatohepatitis in both cultured hepatocytes and mice. Gastroenterology 138, 694–704. 19818781 10.1053/j.gastro.2009.09.058

[30] Houstis N , Rosen ED and Lander ES (2006) Reactive oxygen species have a causal role in multiple forms of insulin resistance. Nature 440, 944–948. 16612386 10.1038/nature04634

[31] Anderson EJ , Lustig ME , Boyle KE , Woodlief TL , Kane DA , Lin CT , Price JW 3rd , Kang L , Rabinovitch PS , Szeto HH *et al* (2009) Mitochondrial H_2_O_2_ emission and cellular redox state link excess fat intake to insulin resistance in both rodents and humans. J Clin Invest 119, 573–581. 19188683 10.1172/JCI37048 PMC2648700

[32] Rains JL and Jain SK (2011) Oxidative stress, insulin signaling, and diabetes. Free Radic Biol Med 50, 567–575. 21163346 10.1016/j.freeradbiomed.2010.12.006 PMC3557825

[33] Bruce CR , Carey AL , Hawley JA and Febbraio MA (2003) Intramuscular heat shock protein 72 and heme oxygenase‐1 mRNA are reduced in patients with type 2 diabetes: evidence that insulin resistance is associated with a disturbed antioxidant defense mechanism. Diabetes 52, 2338–2345. 12941774 10.2337/diabetes.52.9.2338

[34] Nishio Y , Fujino M , Zhao M , Ishii T , Ishizuka M , Ito H , Takahashi K , Abe F , Nakajima M , Tanaka T *et al* (2014) 5‐Aminolevulinic acid combined with ferrous iron enhances the expression of heme oxygenase‐1. Int Immunopharmacol 19, 300–307. 24530569 10.1016/j.intimp.2014.02.003

[35] Ndisang JF , Zhao W and Wang R (2002) Selective regulation of blood pressure by heme oxygenase‐1 in hypertension. Hypertension 40, 315–321. 12215473 10.1161/01.hyp.0000028488.71068.16

[36] Vanella L , Li M , Kim D , Malfa G , Bellner L , Kawakami T and Abraham NG (2012) ApoA1: mimetic peptide reverses adipocyte dysfunction in vivo and in vitro via an increase in heme oxygenase (HO‐1) and Wnt10b. Cell Cycle 11, 706–714. 22306989 10.4161/cc.11.4.19125 PMC3318105

[37] Hou J , Cai S , Kitajima Y , Fujino M , Ito H , Takahashi K , Abe F , Tanaka T , Ding Q and Li XK (2013) 5‐Aminolevulinic acid combined with ferrous iron induces carbon monoxide generation in mouse kidneys and protects from renal ischemia‐reperfusion injury. Am J Physiol Renal Physiol 305, F1149–F1157. 23904222 10.1152/ajprenal.00275.2013

[38] Shimamura Y , Horinouchi I , Matsuda S , Tsuchiya K , Miyanari S , Kobayashi Y , Kuwahata M and Kido Y (2011) Possibility of 5‐aminolevulinic acid for nutritional supplement; Suppression of visceral fat accumulation in rats In Aminolevulinic Acid: Science, Technology and Application (Okura I and Tanaka T, eds), pp. 109–116. SBI ALApromo, Tokyo Institute of Technology Press, Tokyo.

[39] Jenkins DJ , Wolever TM , Ocana AM , Vuksan V , Cunnane SC , Jenkins M , Wong GS , Singer W , Bloom SR , Blendis LM *et al* (1990) Metabolic effects of reducing rate of glucose ingestion by single bolus versus continuous sipping. Diabetes 39, 775–781. 2191884 10.2337/diab.39.7.775

[40] Perez MH , Rodriguez BL , Shintani TT , Watanabe K , Miyanari S and Harrigan RC (2013) 5‐Aminolevulinic acid (5‐ALA): analysis of preclinical and safety literature. Food Nutr Sci 4, 1009–1013.

[41] Kitamura T (2013) The role of FOXO1 in β‐cell failure and type 2 diabetes mellitus. Nat Rev Endocrinol 9, 615–623. 23959366 10.1038/nrendo.2013.157

[42] Weir GC and Bonner‐Weir S (2013) Islet β cell mass in diabetes and how it relates to function, birth, and death. Ann N Y Acad Sci 1281, 92–105. 23363033 10.1111/nyas.12031 PMC3618572

[43] Jung HS , Chung KW , Won Kim J , Kim J , Komatsu M , Tanaka K , Nguyen YH , Kang TM , Yoon KH , Kim JW *et al* (2008) Loss of autophagy diminishes pancreatic beta cell mass and function with resultant hyperglycemia. Cell Metab 8, 318–324. 18840362 10.1016/j.cmet.2008.08.013

[44] Dailey MJ and Moran TH (2013) Glucagon‐like peptide 1 and appetite. Trends Endocrinol Metab 24, 85–91. 23332584 10.1016/j.tem.2012.11.008 PMC3594872

[45] De Siervi A , Vazquez ES , Rezaval C , Rossetti MV and del Batlle AM (2002) Delta‐aminolevulinic acid cytotoxic effects on human hepatocarcinoma cell lines. BMC Cancer 2, 6. 11914144 10.1186/1471-2407-2-6 PMC101407

[46] Onuki J , Chen Y , Teixeira PC , Schumacher RI , Medeiros MH , Van Houten B and Di Mascio P (2004) Mitochondrial and nuclear DNA damage induced by 5‐aminolevulinic acid. Arch Biochem Biophys 432, 178–187. 15542056 10.1016/j.abb.2004.09.030

[47] Fuchs J , Weber S and Kaufmann R (2000) Genotoxic potential of porphyrin type photosensitizers with particular emphasis on 5‐aminolevulinic acid: implications for clinical photodynamic therapy. Free Radic Biol Med 28, 537–548. 10719235 10.1016/s0891-5849(99)00255-5

[48] Bednarz N , Zawacka‐Pankau J and Kowalska A (2007) Protoporphyrin IX induces apoptosis in HeLa cells prior to photodynamic treatment. Pharmacol Rep 59, 474–479. 17901578

[49] Li Q , Wang X , Zhang K , Li X , Liu Q and Wang P (2013) DNA damage and cell cycle arrest induced by protoporphyrin IX in sarcoma 180 cells. Cell Physiol Biochem 32, 778–788. 24080830 10.1159/000354479

[50] Mölzer C , Pfleger B , Putz E , Roßmann A , Schwarz U , Wallner M , Bulmer AC and Wagner KH (2013) *In vitro* DNA‐damaging effects of intestinal and related tetrapyrroles in human cancer cells. Exp Cell Res 319, 536–545. 23246570 10.1016/j.yexcr.2012.12.003 PMC3569715

[51] Su X , Chen Y , Wang X , Wang Y , Wang P , Li L and Liu Q (2014) PpIX induces mitochondria‐related apoptosis in murine leukemia L1210 cells. Drug Chem Toxicol 37, 348–356. 24328896 10.3109/01480545.2013.866135

[52] Juzeniene A , Juzenas P , Iani V and Moan J (2007) Topical applications of iron chelators in photosensitization. Photochem Photobiol Sci 6, 1268–1274. 18046481 10.1039/b703861e

[53] Juzeniene A , Iani V and Moan J (2013) Clearance mechanism of protoporphyrin IX from mouse skin after application of 5‐aminolevulinic acid. Photodiagnosis Photodyn Ther 10, 538–545. 24284108 10.1016/j.pdpdt.2013.05.008

[54] Reagan‐Shaw S , Nihal M and Ahmad N (2008) Dose translation from animal to human studies revisited. FASEB J 22, 659–661. 17942826 10.1096/fj.07-9574LSF

[55] Ganz T and Nemeth E (2012) Hepcidin and iron homeostasis. Biochim Biophys Acta 1823, 1434–1443. 22306005 10.1016/j.bbamcr.2012.01.014 PMC4048856

[56] Liu S , Brown JD , Stanya KJ , Homan E , Leidl M , Inouye K , Bhargava P , Gangl MR , Dai L , Hatano B *et al* (2013) A diurnal serum lipid integrates hepatic lipogenesis and peripheral fatty acid use. Nature 502, 550–554. 24153306 10.1038/nature12710 PMC4141623

[57] Asher G and Schibler U (2011) Crosstalk between components of circadian and metabolic cycles in mammals. Cell Metab 13, 125–137. 21284980 10.1016/j.cmet.2011.01.006

[58] Bailey SM , Udoh US and Young ME (2014) Circadian regulation of metabolism. J Endocrinol 222, R75–R96. 24928941 10.1530/JOE-14-0200 PMC4109003

[59] Yin L , Wu N , Curtin JC , Qatanani M , Szwergold NR , Reid RA , Waitt GM , Parks DJ , Pearce KH , Wisely GB *et al* (2007) Rev‐erbα, a heme sensor that coordinates metabolic and circadian pathways. Science 318, 1786–1789. 18006707 10.1126/science.1150179

[60] Durgan DJ and Young ME (2010) The cardiomyocyte circadian clock: emerging roles in health and disease. Circ Res 106, 647–658. 20203314 10.1161/CIRCRESAHA.109.209957 PMC3223121

[61] Raghuram S , Stayrook KR , Huang P , Rogers PM , Nosie AK , McClure DB , Burris LL , Khorasanizadeh S , Burris TP and Rastinejad F (2007) Identification of heme as the ligand for the orphan nuclear receptors REV‐ERBα and REV‐ERBβ. Nat Struct Mol Biol 14, 1207–1213. 18037887 10.1038/nsmb1344 PMC2743565

[62] Dioum EM , Rutter J , Tuckerman JR , Gonzalez G , Gilles‐Gonzalez MA and McKnight SL (2002) NPAS2: a gas‐responsive transcription factor. Science 298, 2385–2387. 12446832 10.1126/science.1078456

[63] Yamashita K , Hagiya Y , Nakajima M , Ishizuka M , Tanaka T and Ogura S (2014) The effects of the heme precursor 5‐aminolevulinic acid (ALA) on REV‐ERBα activation. FEBS Open Bio 4, 347–352. 10.1016/j.fob.2014.03.010 PMC4050196 24918048

